# A suite of methods for representing activity space in a healthcare accessibility study

**DOI:** 10.1186/1476-072X-4-24

**Published:** 2005-10-19

**Authors:** Jill E Sherman, John Spencer, John S Preisser, Wilbert M Gesler, Thomas A Arcury

**Affiliations:** 1 Department of Geography, University of North Carolina at Chapel Hill, Chapel Hill, NC, USA; 2 Carolina Population Center, University of North Carolina at Chapel Hill, Chapel Hill, NC, USA; 3 Department of Biostatistics, University of North Carolina at Chapel Hill, Chapel Hill, NC, USA; 4 Department of Family and Community Medicine, Wake Forest University School of Medicine, Winston Salem, NC, USA

## Abstract

**Background:**

"Activity space" has been used to examine how people's habitual movements interact with their environment, and can be used to examine accessibility to healthcare opportunities. Traditionally, the standard deviational ellipse (SDE), a Euclidean measure, has been used to represent activity space. We describe the construction and application of the SDE at one and two standard deviations, and three additional network-based measures of activity space using common tools in GIS: the road network buffer (RNB), the 30-minute standard travel time polygon (STT), and the relative travel time polygon (RTT). We compare the theoretical and methodological assumptions of each measure, and evaluate the measures by examining access to primary care services, using data from western North Carolina.

**Results:**

Individual accessibility is defined as the availability of healthcare opportunities within that individual's activity space. Access is influenced by the shape and area of an individual's activity space, the spatial distribution of opportunities, and by the spatial structures that constrain and direct movement through space; the shape and area of the activity space is partly a product of how it is conceptualized and measured. Network-derived measures improve upon the SDE by incorporating the spatial structures (roads) that channel movement. The area of the STT is primarily influenced by the location of a respondent's residence within the road network hierarchy, with residents living near primary roads having the largest activity spaces. The RNB was most descriptive of actual opportunities and can be used to examine bypassing. The area of the RTT had the strongest correlation with a healthcare destination being located inside the activity space.

**Conclusion:**

The availability of geospatial technologies and data create multiple options for representing and operationalizing the construct of activity space. Each approach has its strengths and limitations, and presents a different view of accessibility. While the choice of method ultimately lies in the research question, interpretation of results must consider the interrelated issues of method, representation, and application. Triangulation aids this interpretation and provides a more complete and nuanced understanding of accessibility.

## Background

Researchers studying healthcare accessibility and utilization have long attempted to understand the influence of geography on individuals' use of healthcare services. Geographic access is usually operationalized as some measure of distance to care. Distance can be measured either from the supply perspective (distance from a clinic or hospital) or from the individual perspective (how far an individual has to travel to a healthcare provider) [1,2], or both [3]. However, these measures typically do not account for differences in individual mobility, spatial habits, and subjective meanings of distance, as well as differences in travel environment. *Activity space*, defined as "the local areas within which people move or travel in the course of their daily activities" [4], is a measure of individual spatial behavior that theoretically accounts for these individual and environmental differences and offers an alternative approach to studying geographic accessibility.

From a methodological standpoint, however, measuring activity space is more data and computationally intensive than distance, and its complexity has resulted in its underutilization. Prior to the introduction of Geographic Information Systems (GIS), approximations of activity space typically made use of Euclidean measures like the Standard Deviational Ellipse (SDE) [5-7], and place-based proxies for household locations (such as zip code centroid). The limited availability and expense of collecting spatially referenced data and the computational burden involved in generating SDEs restricted such studies to small samples. With advances in GIS and increasing availability of spatially referenced data, activity space has become a more viable tool for studying accessibility. These same technological advances enable researchers to develop new measures of activity space that improve on the precision of the SDE and better represent actual spatial behavior [8,9].

Although activity space is not a new concept, few studies have compared alternative methods of measuring activity space. Just as different methods of measuring distance (Euclidean distance, network distance, travel time distance) can yield different information and possibly different conclusions [2], different approaches to calculating activity space may yield different types of information and results [10]. By comparing these measures, we "triangulate" or view accessibility from multiple perspectives, and so arrive at a more nuanced understanding of accessibility [11,12]. Moreover, we develop a better understanding of how the measures used influence the results.

Addressing the interrelated dimensions of representation, method, and application are important for advancing accessibility research [13]. This paper describes five different measures of routine activity space, applies them in a rural mountain region, and evaluates their relative usefulness in the study of geographic access to health services. The five measures are (1) the standard deviational ellipse at 1 standard deviation (SDE1); (2) the standard deviational ellipse at 2 standard deviations (SDE2); (3) the road network buffer (RNB); (4) the 30-minute standard travel time polygon (STT); and (5) the relative travel time polygon (RTT).

### Defining activity space

Activity space has been defined and theorized in different ways by researchers working in various traditions, including medical geography, spatial behavior, time-space studies, planning, travel and transportation studies, and human-environment interactions. Activity space represents the spatial movement component of an individual's day-to-day lived experience [14], and thus "experience of place" [15]. This experience of place is thought to mediate between the role of distance and the distribution of healthcare resources in the perception of healthcare accessibility. Activity space is also described as a measure of an individual's degree of mobility [7], incorporating constraints, needs, preferences and resources for movement. Research in space-time geography has also produced a body of work that uses methods and concepts similar to activity space, but uses different terminology. Kwan's "daily potential path area," for example, is used to measure individual access to urban opportunities [9].

Another perspective on activity space has recently emerged from Geographic Information Science (GISc), largely driven by research in human-environment interactions. One of the main challenges in linking social data to physical (environmental) data is determining how to represent people in space: mapping a person is different from mapping a stationary object, as people are not fixed to a single location. The key challenge in "linking people to pixels" lies partly in the difference between fixed and mobile features [16]. Typically, a person's residence – a single, non-dimensional point – is used to mark an individual's location in space. But as people are mobile, a single fixed point does not adequately represent an individual's location. Because activity space also represents "direct contact between individuals and their social and physical environments"[12], it is a potential solution to this problem. By obtaining locations of routine destinations, a two-dimensional space can be developed to represent a person's location. Once these data are available, however, the challenge becomes how to turn these points into a meaningful representation of activity space, and this can vary depending on the geographical context and the objectives of the research.

### Distance, activity space and accessibility

Distance is related to access and utilization; the farther the distance required to travel, the less likely an individual is to use a service, all else being equal. Distance decay – or the attenuation of a pattern or process with distance – is a well-studied geographical phenomenon [12,17]. However, distance is typically a one-dimensional measure that requires knowing between which nodes to measure. For example, some studies have found a greater propensity for individuals to utilize health services that are near place of employment rather than residence [2,7].

Another limitation of distance is that it does not typically account for individual preferences or other factors that channel movement in a specific direction. Directional bias is related to preferences for a particular place over other places of equal distance due to some perceived quality of the preferred place [12]. Both the distribution of opportunities and individual characteristics are critical factors in determining geographical access and utilization. Because activity space is comprised of directional and temporal components of spatial movement in addition to distance, activity space supplies more information than a distance-only measure by demonstrating point patterns and degree of eccentricity, and is suggestive of how boundaries and transportation networks influence activity patterns. For example, Gesler and Meade's Savannah study found a correspondence between activity space and healthcare-seeking space, suggesting that urban structure was more important than demographic characteristics in influencing activity space [7].

Activity space can also account for what we call "relative distance," or individual tolerances for travel and distance to care. Both absolute distance and travel time can have different subjective meanings [15]. Age, ethnicity, income, social status, and health status and type of care sought all contribute to different distance tolerances [18-21]. Mode of transportation is also clearly important, in that 10 miles to someone with access to an automobile is a different burden than 10 miles to someone without access to a car. Where some tolerate a daily commute of one hour or longer, others may find half an hour to be a disincentive. In rural populations, individuals may expect to routinely travel father distances than their urban counterparts; where some may interpret this as a burden, others may interpret this as a benefit of "getting away from it all" [22,23].

### Measuring activity space

Historically, activity space has been operationalized using the standard deviational ellipse (SDE) [5,6,24]. Analogous to the univariate statistical measure of standard deviation, the SDE is a bivariate statistical measure that provides a comparable estimate of an individual's activity space [6]. Gesler and Albert define it as "an ellipse whose major and minor axes are drawn to represent the magnitude of the minimum and maximum dispersion of a set of points from their mean center" [4]. By mapping daily trips and determining the locations of regular activities, SDEs are calculated based on distance and direction of these locations from the home. Activity frequencies and duration of activity can be used to weight the relative importance of each point [7]. The main limitation of the SDE is that it is an abstract representation of where people go. As a Euclidean measure, it does not account for actual spatial arrangements of geographic or human features. However, the SDE provides a better indicator of individual access than distance alone, and is now relatively easy to generate with available software.

Alternatives to Euclidean representations of activity space are largely network-based measures that utilize road network data to construct paths between points. This approach too has limitations, in that it assumes all movement is channeled by paved roads (unpaved roads are rarely available in commercial road data). Data quality of road network databases varies, and errors in the network, or out-of-date data will induce errors in the activity space. And, unless the actual route or path that a person takes to travel to each destination is known, decisions must be made on whether to connect the activity destinations to the household only or whether to construct paths between all of the destinations. Weighting destinations by frequency of visit is possible, yet requires a degree of arbitrary decision-making instead of the statistically determined weights of the SDE. Yet the network shape that results is a much more realistic representation of the space through which a person travels than the SDE. One example of a network-based approach is Kwan's "daily potential path area," constructed by calculating all the routes connecting the residence and activity nodes and creating a buffer around the nodes and paths [9].

Kwan published a comparative analysis of 30 network-based measures of individual accessibility, distinguishing between integral and space-time measures [10]. Integral measures were those that calculated "cumulative opportunity" based on counting the number of opportunities accessible within a given distance parameter (such as travel time) from the location of a single point (usually residence). Space-time measures such as the "daily potential path area," in contrast, were used to calculate "feasible opportunities" based on accessibility from multiple points representing residence and daily activities.

### Activity space and healthcare accessibility

Providers or services located within the spatial and temporal bounds of a person's routine activity space can be considered more accessible than those located outside the activity space. A service "inside the activity space" is interpreted as one where a person could potentially obtain care with relative ease (not accounting for other dimensions of accessibility), while those opportunities "outside the activity space" are interpreted as requiring the person to deviate from normal routine or expend extra resources to access those opportunities. This fits Joseph and Phillips' (1984) concept of "effective accessibility" which meant, among other things, that the services were available within an individual's space-time budget [25,26], as well as Kwan's "feasible opportunity set" [9,10]. Thus, as a measure, the number of healthcare opportunities inside the activity space is interpreted as an indicator of individual healthcare accessibility.

Both the activity space literature and the space-time geography literature ultimately indicate that, while both the physical and social environments contribute to the spatial structuring of opportunity, individuals do not limit themselves to the "nearest" opportunity. Kwan concluded that spatial accessibility to jobs had no relationship with the length of commute, and questioned the appropriateness of using commuting distance as a measure of job access [9]. The corollary in health services research is that distance to the service actually used may not be an appropriate measure of geographic accessibility, as indeed studies of provider and facility choice, bypassing behavior, and health-seeking behavior have long indicated [27-32]. However, the location of a service actually used relative to activity space may be significant. Nemet and Bailey found that the presence of a respondent's actual provider within the individual's activity space was significantly associated with number of visits [15]. Thus, a provider located within an individual's activity space was "near" and one located outside the activity space was "far," regardless of the absolute distance.

## Methods

### The MAP survey

**Figure 1:**
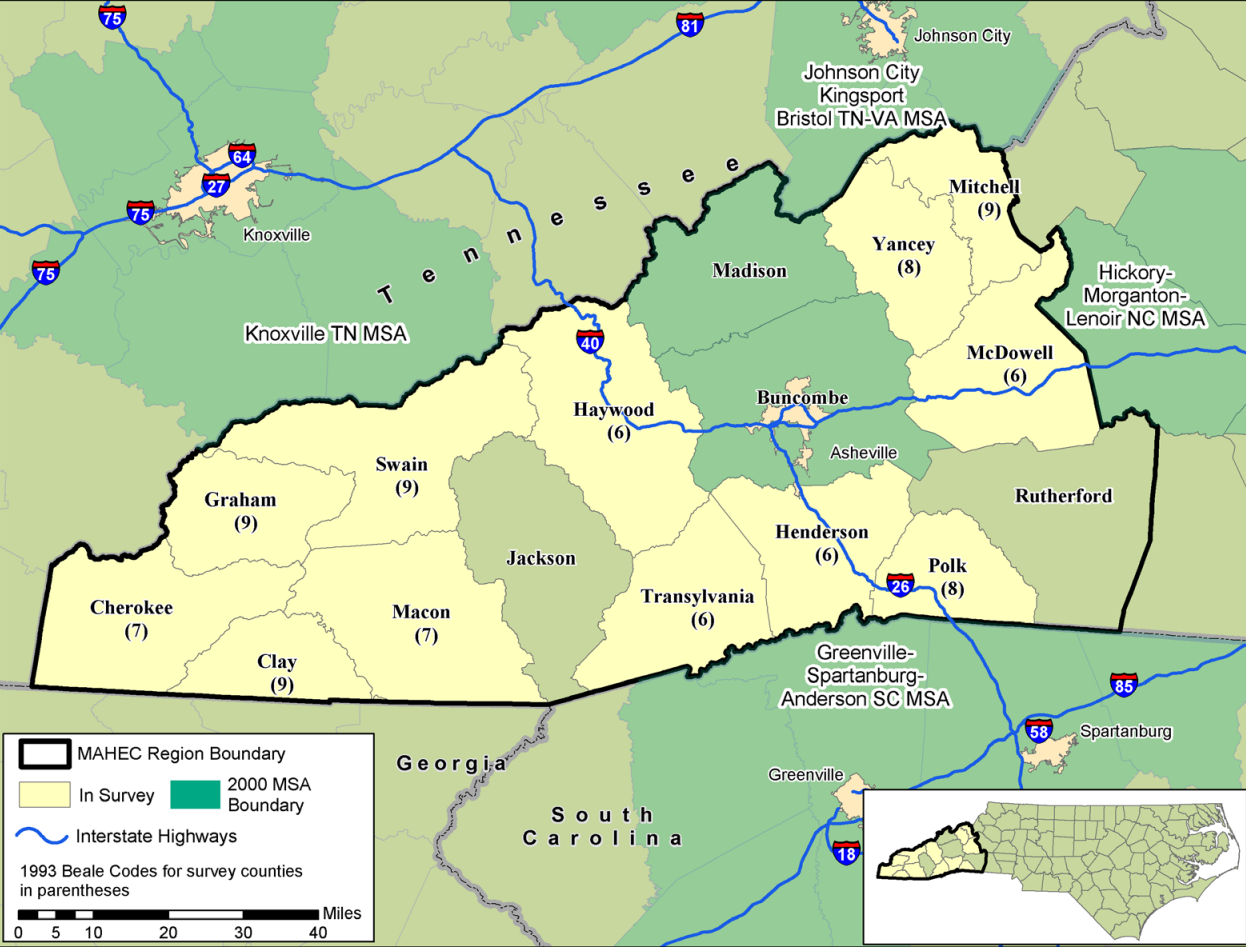
**MAP project study area in western North Carolina**. Twelve rural counties were selected from the Mountain Area Health Education Center (MAHEC) region, based on degree of rurality as measured using 1993 Beale codes. The city of Asheville in metropolitan Buncombe County is the major service center of the region. Interstate highways connect residents of the region with other nearby metropolitan areas in the state, as well as in the bordering states of Virginia, Tennessee, Georgia, and South Carolina.

This research is part of the larger Mountain Accessibility Project, or MAP, which aims to study the effects of rurality and other geographic factors on healthcare accessibility and utilization in a mountain rural region of western North Carolina. The study area consists of 12 counties in the Mountain Area Health Education Center (MAHEC) region, selected to represent the most rural end of the rural-urban continuum as measured using 1993 Beale codes 6–9 (Figure 1). The metropolitan area of Asheville is the major service center of the region; interstate 40 runs east-west through the region, connecting the Asheville and Knoxville MSAs, while I-26 runs south from Asheville to the Greenville-Spartanburg MSA. Over the past decade, the region has seen high rates of retirement, recreation, and natural amenities-related development and in-migration [33-35].

The MAP survey is the primary data source for this analysis. A total of 1,059 adult interviews were completed from June 1999 to January 2000, yielding a rich dataset consisting of individual- and household level data, including variables on healthcare accessibility and utilization; sociodemographic characteristics; transportation and spatial behavior; health status and health behavior; and cultural and attitudinal variables. Spatial data were collected for household locations, routine activity destinations, and locations where respondents reported obtaining health services during the year preceding the interview. The study protocol was approved by the Institutional Review Boards of the University of North Carolina at Chapel Hill, Wake Forest University School of Medicine, and Research Triangle Institute. Details of the study design are reported elsewhere [36,37].

Although the objectives of this paper are primarily methodological, the characteristics of the study area exert important influences on the results. Apart from the physical features and transportation networks noted above, the project documented the finding that the region is well supplied with primary care services [38]. While there are spatial variations in service supply, we were unable to identify sub-regions or subpopulations where the geographic accessibility of primary care services failed to meet federal guidelines. On the one hand, this implies that we are unable to compare the performance of these measures in an environment with poor availability and accessibility. On the other hand, this context allows us to compare these measures to a kind of "gold standard" of primary care accessibility.

### Collecting activity data

Activity spaces are constructed from "the subset of all locations within which an individual has direct contact as a result of his or her day-to-day activities" [12]. Three types of locational variables were collected in this survey: residence, routine activity destinations, and healthcare destinations. The primary node in each activity space is the location of residence. Interviewers were trained in the use of GPS receivers and recorded the latitude and longitude of the household at the time of the survey. For households where valid GPS coordinates were not collected, two backup methods of georeferencing the residence location were utilized. First, the project produced maps of the entire study area divided into grid cells of one square kilometer; interviewers were to record the coordinates of the map grid cell in which the residence was located, and coordinates of the grid cell centroid were used to georeference the household. Where neither valid GPS nor map coordinates were recorded, street addresses provided the option of address geocoding using a commercially available database. All households were thus able to be georeferenced.

The other nodes in the activity space were the locations of the routine activity destinations. Using the gridded maps, respondents were asked to identify the locations of where they usually went for a total of 34 possible routine activities, including grocery shopping; to pick up items from a convenience mart; buy gasoline; get the car serviced; clothes or retail shopping; visit friend or family member; eat out; to see movies; go to church or other religious activities; play sports or exercise; watch sports; bank; buy stamps and send letters and packages; go to school; work; and any other common activity not specifically prompted. Interviewers were to prompt respondents for up to two locations per activity type.

Respondents also reported the locations of healthcare providers and facilities visited in the year preceding the interview. Regardless of the frequency of visits, these health destinations were not treated as "routine activity destinations" and were not used in constructing activity spaces. The goal was to compare the degree of overlap or correspondence between routine activity spaces (e.g. non healthcare) and healthcare destinations. Locational data on healthcare destinations were used to test whether the different measures of activity space captured the actual healthcare destination for each respondent. We suggest that the best measure of routine activity space for studying healthcare accessibility will effectively capture not only potential healthcare opportunities, but also the locations of services actually used.

In order to construct the activity space measures, it was necessary to place spatial and temporal bounds on activity destinations reported by respondents. We spatially limited the destinations to those points located within North Carolina (inside or outside of the study area) and the neighboring states of Virginia, Tennessee, Georgia, and South Carolina. This spatial bounding was necessary because some respondents reported traveling great distances on a routine basis. While these destinations may have very important impacts on respondents' lives, we interpreted them as "non-local" activities that did not interact with the local healthcare environment. Healthcare destinations were similarly bounded.

Frequency of activity is also an important data component of activity space. The frequency of "daily" or "routine" has not been defined in any consistent manner in previous studies. Nemet and Bailey defined their activity space as the spatial extent of an individual's weekly activities, but response categories on the questionnaire were "never," "less than once a week," and "more than once a week" which were then weighted in the calculation of the SDE [15]. Kwan used a detailed two-day diary to collect daily activity destinations in sequence [9]. In the MAP survey, respondents were asked to indicate frequency in terms of the number of times per day, week, month or year that they visited each routine activity destination, and these frequencies were converted into a fraction of 365. For this analysis, we limited the construction of routine activity spaces to those activities with a frequency of 12 or more (at least once a month).

Of 1,059 completed interviews, 1,047 respondents reported at least one activity destination with a frequency of at least once a month. Ten of these reported only one destination, and 20 reported only two destinations (in nine of these cases, the destinations had the same coordinates, resulting in effectively only one point). Generating the two axes of an ellipse requires at least three unique points (including the household location); thus, for 19 cases, we had insufficient data to generate ellipses. To avoid dropping these cases and inducing bias toward larger activity spaces, we constructed 1 km circle buffers around single unique points, and likewise connected 2-point cases with a 1 km linear buffer around the two points. These procedures only affected the two SDE measures.

### Other data sources

Two additional data sources were required for this study. First, in order to construct the network-based measures (RNB, STT, RTT), a road network database was required. The road network for the region was obtained from ESRI's StreetMap 2000 product [39]. In addition to providing a spatial representation of the road network for the region, it also contains the Census Feature Class Codes (CFCC) for the road segments. CFCCs are standardized descriptions of the type of road and can be used, among other purposes, to assign speed limits to roads; these speed limits are used in the calculation of travel time. For this project a standardized set of speed limits by CFCC provided by ESRI were used.

The third data source provided the locations for healthcare practitioners and facilities in western North Carolina. A database provided courtesy of McMillan & Moss Research, Inc., was constructed from a 2000 survey using GPS receivers to collect the spatial locations of all healthcare delivery sites in the region [40]. From this dataset we subset the locations where primary care services were available. The database did not provide the kind of data that would enable us to classify a service delivery point based on the type of service or facility (private practitioner, clinic, hospital), health workforce (number or type of providers per location), or capacity (e.g. number of patients in practice). However, the point locations do represent "primary care opportunities," or PCOs, where a respondent could potentially seek care.

Another limitation of this dataset is that it includes only 15 counties within the MAHEC service region. All study counties shared a border with counties not covered by the database. Patients, of course, do not necessarily observe county or state borders when seeking health services; in our survey, 48% percent of reported health destinations were in a different county than county of residence. Respondents could potentially have more primary care opportunities available within their activity space than we were able to count because of services available on the other side of the border. This "edge effect" occurs when the administrative boundaries of a dataset artificially bound what is in reality a continuous surface [2]. The project did make efforts to obtain locational data for health services in bordering counties in North Carolina, Tennessee, Georgia and South Carolina, however comparable data were not obtainable. An assessment of the extent of the edge effect revealed that, out of 2,872 reported health destinations (multiple destinations per respondent were possible), 13% were outside the counties covered by the McMillan & Moss database, with 5% out of North Carolina. Half of these out-of-state destinations (n = 66) were reported by residents of Polk County, which has a high rate of commuting to work to the Greenville-Spartanburg MSA in South Carolina. Clay and Cherokee counties accounted for 24% and 17% of out-of-state health destinations, with negligible numbers attributed to the other counties. Thus, while the boundary crossing problem potentially affects residents of all counties in the study area (resulting in an underestimate of availability of health services), results of the survey data suggests that the problem affects only a small proportion of respondents in 3 of the 12 counties.

To determine the routes between the points of the activity space and perform network calculations, points need to be located on the road network. Neither the household coordinates nor the activity destination coordinates were necessarily on the road network. Some households could be a considerable distance from the road network, for example, if they lived on an unpaved road or had a long driveway. Use of grid centroids to determine locations may have placed some points off the network. Unknown errors in the road network data could also introduce a gap between the network and the location. Points not located close enough to a road network segment were "snapped," or moved to the nearest segment of the road network by the GIS. This new snapped location was used when calculating the network-based travel time polygons and road buffers.

Snapping is a standard procedure in GIS, but it does induce a small amount of error. The mean Euclidean distance that household points were snapped was 131 meters, with the maximum being 1.2 kilometers. The mean distance the activity destination points were snapped was 154 meters, with the maximum being 5.2 kilometers from its original location. We concluded that snapping had a minimal impact on the distance estimates. This snapping error was calculated to assess the impact of snapping on the travel estimates; they cannot be used to adjust travel distance or time to "account for snapping error" because of the difference between Euclidean and road distance, and because snapping could move a point closer or farther from the actual travel path. Because it was not possible to determine exactly how snapping affected each household or activity location, no "adjustment" to travel distance/time was made to counteract the effect of snapping; any such procedure would have induced more error.

### Constructing the activity space measures

In this section we will describe how each measure is constructed, using five measures of the activity space of a single respondent to illustrate. At the same time we will describe the hypothesized advantages and disadvantages of each measure prior to analyzing and interpreting the results.

#### The standard deviational ellipse (SDE)

For the routine activity SDE, the location of the respondent's residence and each of the routine activity destinations was mapped in the GIS. Each point was weighted based on the number of times per year the respondent went to that destination, with the respondent's residence given a value of 365, assuming the respondent was there every day. Once the spread of points was mapped, an ellipse was generated using the formula found in the spatial statistics program CRIMESTAT [41]. The standard deviation of the distances between each point and the mean center are calculated for the X direction and the Y direction. This distance is used as the major and minor axis of the ellipse [41].

Because of the structure of the data table, it was necessary to write a script which could accommodate the data file, rather than relying on a pre-existing application such as CRIMESTAT to generate the ellipses. The script developed by the authors provides the following output: the area of the ellipse, the lengths of the ellipse's X and Y axes, its theta angle (the angle between the major axis and the horizontal or X axis), and the location of the weighted mean center of all points. Additionally a graphic representation of the ellipse was generated to be included in the GIS.

Because the literature is inconsistent on the use of one or two standard deviations in generating the ellipses, we chose to do both for comparative purposes. The SDE at one standard deviation (SDE1) contains approximately 68% of the points within the boundary of the ellipse, while the SDE at two standard deviations (SDE2) encompasses approximately 95% of the points (if all destinations are given equal weight; weighting destinations based on frequency of visit will alter these proportions).

**Figure 2:**
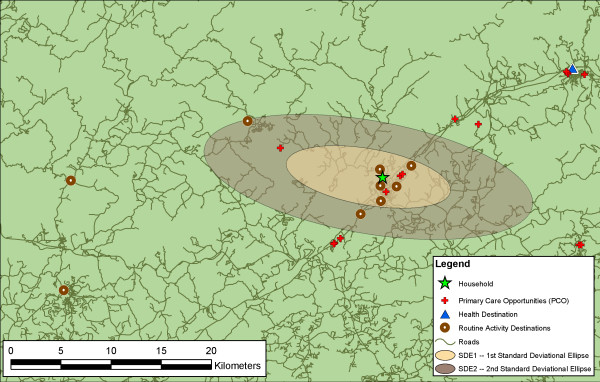
**Example of the one and two standard deviational ellipse (SDE1, SDE2) measures**. The figure shows the activity space of a single respondent as represented by the two Euclidean measures. The activity destination furthest from the household is the respondent's place of employment. The high frequency of trips to the workplace exerts a strong influence on the shape and orientation of the ellipses. Note, however, that each ellipse encompasses large areas without any activity destinations. Also note that there is no route between the household and the workplace that lies within the boundaries of either ellipse. Finally, while there are 3 and 4 primary care opportunities within the SDE1 and SDE2 respectively, neither ellipse captures the respondent's actual health destination.

To illustrate, Figure 2 shows the SDE of one respondent, at 1 and 2 standard deviations, along with the residence and activity destinations. This respondent has a total of 15 routine activity destinations; however, only nine unique destinations in terms of location are visible because of shared coordinates. Also shown are the primary care opportunities in the region. For this respondent, three opportunities lie within the SDE1, and four within the SDE2, and are suggestive of reasonably good access to primary care services. However, this respondent's actual health destination is not inside either ellipse.

#### Road network buffer (RNB)

The activity space of the RNB is essentially the area around the roads that an individual is likely to travel from home to all destinations; this measure is the closest to Kwan's (1998) time-space measures (although the temporal component is treated quite differently). Within the GIS, the shortest road distance between the respondent's household and each destination was calculated using the ArcView 3.3 Network Analyst extension [42]. Then a buffer was calculated around each route to create a "polygon" in a GIS. Routes were aggregated into a single shape for each respondent using the dissolve function in ArcView to erase the boundaries between buffered routes. A program written in Avenue (ArcView's programming language) was used to automate the process.

The size of the buffer was set to 1 km, meaning that an area of 1 km on each side of the road is encompassed in the polygon. The rationale for choosing a buffer size of 1 km was that (a) given the use of the centroids of 1 km map grid cells to geocode the activity destinations, we have approximately 1 km of error built into the distance between two points, and (b) 1 km off the path would not be considered an additional burden in terms of travel distance, even if walking, for most people.

There are limitations in the calculation of the RNB measure. First, it requires the use of a GIS to calculate, and requires an adequate road dataset. The quality of the measure is dependent on the quality of the road data. Also, the method chosen to calculate the RNB was by calculating the shortest route (a function of road segment length) between a respondent's house and each activity destination, based on the assumption of the most likely route. However, it is possible the respondent may have chosen a different route for their journey. In the absence of data on the actual paths or routes the individual travels to each activity destination, any number of choices could be made. Kwan's 2-day travel diaries included data on trip-chaining and times at each location, and she chose to calculate the paths between each node [9]; however, these paths are limited to activities in a two-day period and may include non-routine as well as routine activities, thus limiting their generalizability to a longer time frame or broader definition of "routine."

**Figure 3:**
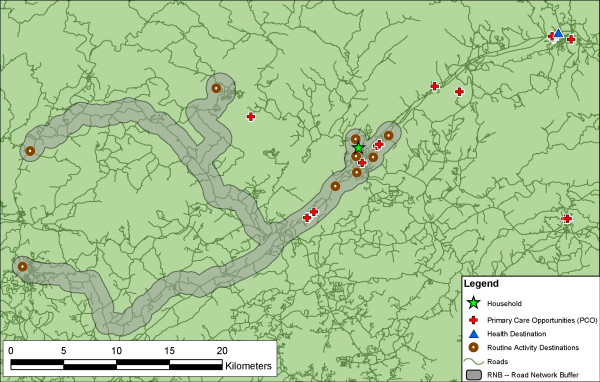
**Example of the road network buffer (RNB) measure**. This is the activity space of the same respondent as represented using the RNB measure. This activity space captures a different set of primary care opportunities based travel patterns structured by road networks. It also eliminates the excess space captured by the SDEs. However, the actual health destination is still outside of the activity space.

In Figure 3, we show the RNB activity space for the same respondent as shown in Figure 2. The shape, area, and extent of the activity space using the RNB are quite different from the SDE, illustrating one of the major limitations of the SDE. For this respondent, not only is there a different number of primary care SDPs inside the activity space (5), but also the set of primary care opportunities identified as "accessible" within each measure – although overlapping – are different (this is not necessarily true for all respondents). Rather than hypothetical access, the RNB representation of activity space identifies the actual SDPs that a person presumably passes. By comparing the points that lie within the buffer to the points that the respondent actually used, we could construct an index of bypassing, although that is beyond the scope of this paper.

Of the five measures, the RNB might be considered the best representation of the spatial movement component of a respondent's activity space, because the area is limited to the likely routes and locations that a person travels. It is descriptive of what the person actually does, not what the person could or should do. The RNB measure is also one of only two measures presented here that encompass 100% of the activity destinations. This measure is the most useful for understanding issues of bypassing and provider/facility choice.

#### Standard travel time polygon (STT)

Like the RNB, the two travel time polygon measures are based on network calculations. They are constructed by determining a travel time limit, traveling outward from the household location to that limit on all roads within the network leading away from the household, and bounding the area to create a polygon. For the STT, a GIS was employed to determine how far each respondent could travel from their house in thirty minutes. Every road available to them, within thirty minutes, was included in this analysis. This procedure was performed in ArcView 3.3 using the "service area" command in the Network Analyst Extension. A polygon was then created which encompassed all roads within that 30-minute threshold and this serves as the STT activity space used for analysis. The standard of 30 minutes was chosen because of its use in other studies of healthcare accessibility and distance [1,2,43], and because it serves as the federal guideline for maximum distance to primary care under the Health Professional Shortage Area guidelines [44].

The STT is thus based strictly on location of residence and the road network; the routine activity destinations are not used in the construction of this measure. Because the size and shape of the polygon is independent of the activity destinations, the measure does not represent the actual activity space reported by respondents, but is a measure of "potential" activity space because it captures where the person could go within a specified level of effort. It is a normative representation of activity space comparable to Kwan's integral measure of "cumulative opportunity" [10].

**Figure 4:**
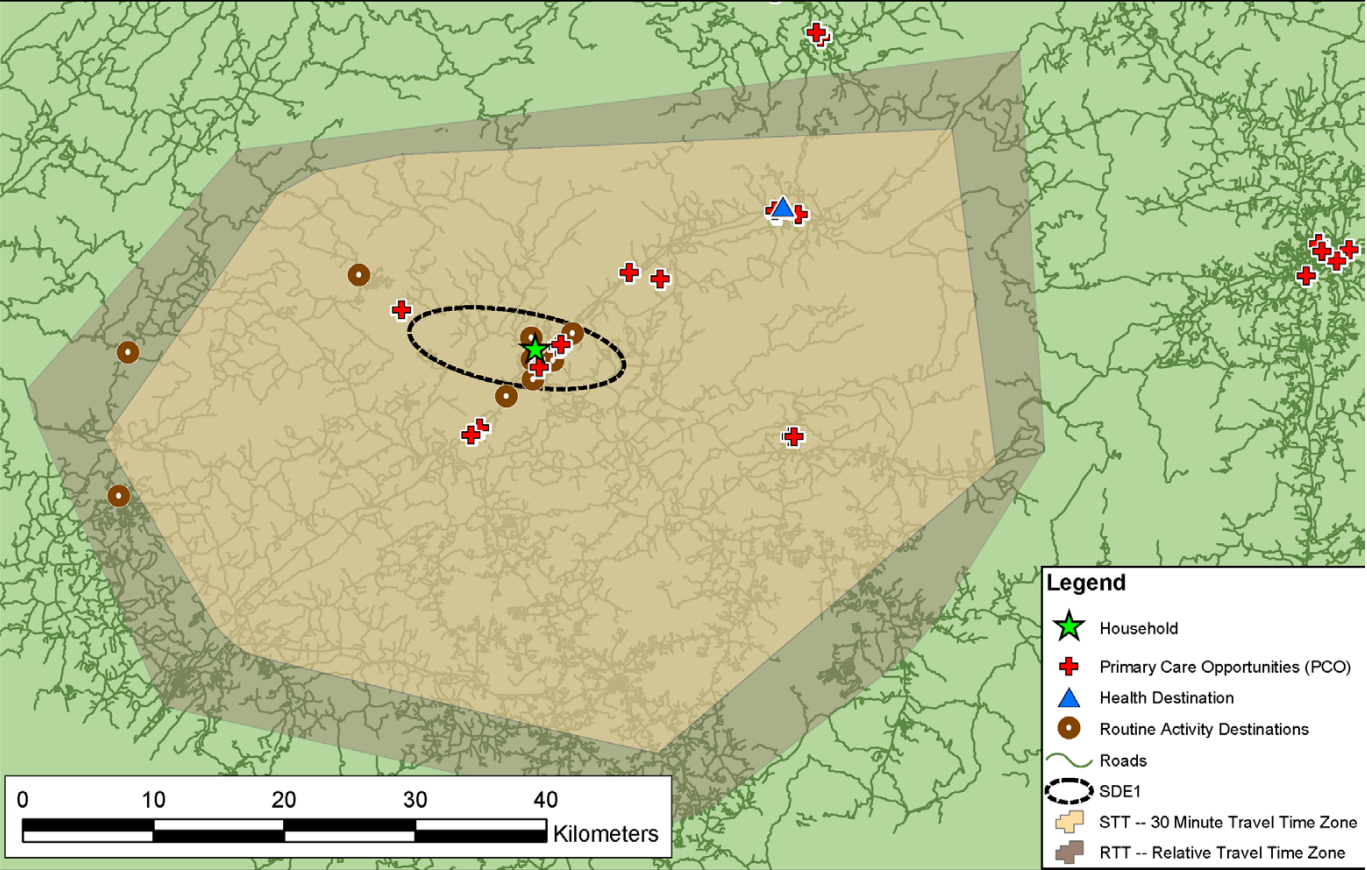
**Example of the standard travel time polygon (STT) and relative travel time polygon (RTT) measures**. For the same respondent, the two potential activity spaces as represented by travel time polygons. In this example, the RTT is larger than the 30-minute STT, indicating that the respondent routinely travels farther than 30 minutes (two activity destinations lie outside of the STT polygon). The smaller STT does capture the actual health destination, however, along with numerous primary care opportunities.

Figure 4 displays the STT measure for the example respondent. The scale of the travel time measures greatly exceeds the SDE and RNB measures, as do their areas. The area of the STT is 2384 km^2^, in comparison with 73 km^2 ^and 293 km^2 ^for the SDE1 and SDE2, and 187 km^2 ^for the RNB measures. In this example, 13 of 15 routine activity destinations were captured by the STT, indicating that the respondent travels further than 30 minutes in reaching two routine activity destinations. This respondent has 11 primary care opportunities within 30 minutes, clearly meeting federal guidelines for accessibility to primary care. Importantly, this respondent's actual health destination is also within the 30-minute travel time polygon. This is not necessarily true for all respondents.

#### Relative travel time polygon (RTT)

This measure was calculated in the same way as the STT, however, the travel time threshold was individually determined and therefore different for each respondent. To determine the relative travel time threshold, the GIS used the most direct route (in travel time) between a respondent's home and each of their activity destinations. In order to determine the most direct route, the GIS traversed all possible routes between the household location and activity destination, and then determined the route with the shortest travel time. The travel time from the household to each routine activity destination was calculated, and the longest time for that respondent was used to set the relative threshold distance and generate the relative travel time polygon (again, the process was automated using an Avenue script). For instance, the travel time to the farthest destination in this respondent's activity space was 36 minutes. The GIS determined how far that respondent could travel in 36 minutes in all directions; this maximum extent was used to create the RTT polygon (Figure 4).

The shape of the RTT is similar to the STT, however it is a little bit larger for this respondent (the reverse is equally possible). The RTT by definition captures all 15 of the routine activity destinations, and is similar to the RNB in this characteristic. In this example, the RTT has the largest area of the activity space measures at 3351 km^2^, although this is unlikely to be true for all or even most respondents. In this case, the extra distance did not capture any more opportunities than the STT, but like the STT it did capture the actual health destination.

Conceptually, this measure is a hybrid of Kwan's integral cumulative opportunity measure and the space-time measures. It is constructed like the integral measure based on proximity to a single location; however, the threshold is empirically determined for each individual based on the routine activity destinations. We suggest that the strength of this measure is it takes into account the amount of travel burden that is acceptable to the individual while not excluding opportunities that may be equally accessible. Like the STT, it represents a potential or normative situation; a person could go to a destination within these bounds without undue travel burden, even if that person normally does not.

There are some limitations in the use of calculating travel time that apply to both the STT and the RTT. The first is in the use of standardized speed limits. In actuality, the speed limits of the roads may not match the standard; however determining the true speed limits of the roads in the study area exceeded the time and resource restraints of the project. The second limitation is that not everyone travels at the designated speed limit, so actual respondent travel times will likely vary. Additionally, the travel time calculations do not take into account variables such as traffic lights, one-way streets, or traffic congestion, all of which can affect the time a respondent needs to travel to the destination. Another major limitation of this approach is that it assumes the same mode of transportation is available for all routes; for individuals dependent on public transportation, this is likely to be a false assumption.

### Data analysis

The analysis had three main objectives. (1) The first was to explore the similarities and differences between the measures using descriptive statistics; means and medians of area are presented along with factors thought to influence the size and shape of activity space. (2) Second, we compare the measures using indicators of accessibility constructed from the number of primary care opportunities located inside the activity space. (3) Finally, we assess how well each measure captures a respondent's actual health destination, and test the association between success at capturing a health destination and the area of the activity space, as well as the number of primary care opportunities. This is based on the assumption that the best measure of activity space for examining healthcare accessibility will be one that also successfully models where people actually go for health services. Statistical analysis was performed using Stata 8 [45].

Three main variables were constructed from each of the five activity space measures. First, area or the size of the activity space was calculated in square kilometers. The distribution is highly skewed towards the higher end of the range (e.g,. right-tailed). For use in analyses that require a linear measure, the measure is transformed from the quadratic scale to the linear scale by taking the square root of the area and truncated at the 2^nd ^and 98^th ^percentiles to minimize the effect of outliers.

The second variable is a count of the number of primary care opportunities (PCOs) located inside each respondent's activity space; ArcView's point-in-polygon procedure was used to calculate this variable. Like the area measure, the range of the number of primary care opportunities was highly skewed, and this measure also was transformed using the square root transformation.

The third variable was a dichotomous (no/yes) variable indicating whether an actual health destination was inside the activity space for each measure. Seventy-seven percent (n = 807) of respondents reported visiting an allopathic provider in the year preceding the interview; although many reported multiple providers and locations, we used only the location of the first reported provider. No significant differences in area or number of PCOs were found between those who reported a health destination and those who did not, for any of the activity space measures.

In order to better understand the differences between the measures, we examined the associations between the area of the activity space and a number of potential influences: number of routine activity destinations, maximum travel time to a routine destination (the threshold used to determine the extent of the RTT), travel time to nearest PCO, and proximity to primary roads. Both the number of destinations and the maximum travel time are components of the SDE1, SDE2, RNB and RTT measures, but not the STT measure. Since these were not used in the construction of the STT measure, we hypothesized that the road network characteristics were the primary determinant of the area of the STT. Respondents who lived near primary roads – roads that are assigned high average speeds – were likely to have the largest STT areas, and indeed, all of the network measures might be influenced by proximity to primary roads. To test this, we created a dichotomous (no/yes) variable, to indicate whether respondents lived within 5 km of a primary road. A 5 km buffer was constructed around the primary roads in the region, and the point-in-polygon procedure was used to determine which households were located inside the buffer.

### Modeling individual accessibility using activity space

We compared three accessibility measures across the five representations of activity space: the proportion of respondents with at least one PCO inside the activity space; the mean/median number of primary care opportunities inside the activity space; and the proportion of respondents whose actual health destination was inside the activity space. Finally, to further explore the relationship between the area of activity space and healthcare accessibility, a logistic regression model was used to test the relationship between the area of the activity space and the presence of an actual health destination inside the activity space. Because the difference in range of area values between the measures, areas were normalized using z-scores and centered around the mean in order to place the five measures on a comparable scale. Results were obtained using Stata's logistic procedure, incorporating the cluster option to control for design effects.

## Results

As expected, the area of the activity space varied dramatically by measure (see Table 1). The RNB activity space had the smallest mean area (107 km^2^), while the SDE1 had the smallest median area (71.5 km^2^). The area of the SDE2 measure was 4 times the SDE1 measure. The relative travel time polygon (RTT) had the highest mean value, while the 30-minute standard travel time polygon (STT) had the largest median area. For a majority of respondents (60%), the area of the STT was larger than the RTT, indicating that the maximum travel time of 30 minutes to a primary care provider could potentially require a large number of residents in this area to travel further than they routinely travel to obtain care.

**Table 1:** Area of activity space and proximity to primary roads for the five measures. Means and medians reported for untransformed measures. SDE1 = standard deviational ellipse at one standard deviation; SDE2 = standard deviational ellipse at 2 standard deviations; RNB = road network buffer; STT = standard travel time polygon; RTT = relative travel time polygon.

	**SDE1**		**SDE2**		**RNB**		**STT**		**RTT**	
	Mean	Median	Mean	Median	Mean	Median	Mean	Median	Mean	Median

										
**Area **(km^2^) (n = 1047)	182.2	71.5	728.7	286.0	107.0	95.7	2301	2160	2845	1425
**Area and residential proximity to primary roads:**										
More than 5 km away from primary road	233.2	120.6	932.8	482.5	130.5	115.8	1333.2	1219.9	3256.0	2141.1
Within 5 km of a primary road	175.5	64.9	702.1	259.6	103.8	92.4	2432.6	2226.5	2789.7	1344.2

### Correlations among area measures

**Figure 5:**
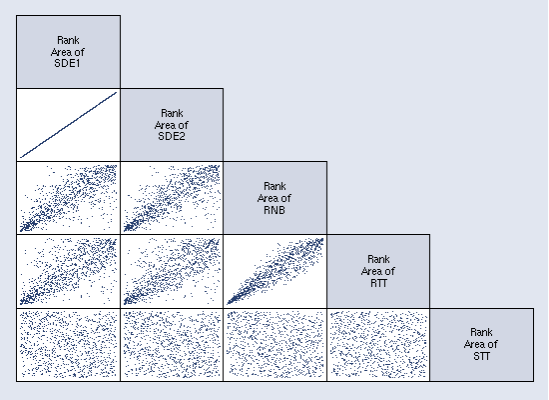
**Scatterplot matrix of rank-transformed areas of the five activity space measures**. Visual representation of the correlation matrix showing the Spearman's rank correlations between area measures. The matrix shows the perfect correlation between the SDE1 and SDE2, the strong correlation between RNB and RTT, and the lack of correlation between the STT and the other measures.

Table 2 shows the correlation matrix of the areas for all five measures. The areas of the SDE measures were perfectly correlated; latter analyses will illustrate their differences. The RNB and RTT measures were strongly correlated (0.924) despite the drastically different size and shape of the measures. The RNB had the strongest correlation with the other measures. The STT was weakly, negatively correlated with the other four measures. The ranges of values suggest that each measure maps a different activity space and will capture different, perhaps complementary, information. The scatterplot matrix in Figure 5 provides a visual representation of the correlation matrix.

**Table 2:** Correlations between areas of the five measures, and between the measures and potential determinants. Spearman's rank correlation. SDE1 = standard deviational ellipse at 1 standard deviation; SDE2 = standard deviational ellipse at 2 standard deviations; RNB = road network buffer; STT = standard travel time polygon; RTT = relative travel time polygon.

	**SDE1**	**SDE2**	**RNB**	**STT**	**RTT**
**Correlations between the areas of the activity space measures**					
SDE1	1.000				
SDE2	1.000	1.000			
RNB	0.782	0.782	1.000		
STT	-0.113	-0.113	-0.153	1.000	
RTT	0.709	0.709	0.924	-0.104	1.000
					
**Correlation between area and the number of reported activity destinations**	0.355	0.355	0.479	0.068	0.396
					
**Correlation between area and maximum travel time to a routine destination**	0.686	0.686	0.900	-0.362	0.954
					
**Correlation between area and travel time to nearest primary care opportunity**	0.277	0.277	0.188	-0.536	0.099
					
**Correlation between area and residence located within 5 km of a primary road**	-0.154	-0.154	-0.121	0.385	-0.085

### Determinants of area

Respondents reported a mean and median of 9 routine activity destinations, with a minimum of 1 and a maximum of 22; these values were approximately normally distributed. The area of the RNB activity space had the strongest correlation (.479) with the number of activity destinations (Table 2). Since the STT was dependent only on the household location, it had the weakest correlation (.068).

The mean and median travel times to the farthest activity destination were 28.6 and 24.7 minutes respectively, with a minimum of .39 and a maximum of 175 minutes. Because the threshold travel time of the RTT is the same as this time, the two measures were near perfectly correlated. Three other measures also had strong positive correlations with the activity travel time maximum: SDE1 and SDE2 (.686), and the RNB (.900). Again the exception, the STT had a moderate negative correlation (-.362).

None of the area measures were even moderately correlated with travel time to the nearest PCO, except for the STT, which had a moderate negative correlation (-.536). Because the STT measure behaved so differently from the other measures, we hypothesized that location within the road network was the primary determinant of the area of the STT polygon. We then tested the association between area and residential proximity to primary road (defined as within 5 km of a primary road). Only the STT area measure was positively correlated (.385); the other four measures had weak negative correlations. For the STT measure, the median area for those living near primary roads (2227 km^2^) was larger than for those living more than 5 km from primary roads (1220 km^2^), indicating that respondents who live near primary roads have the largest potential activity space at a given travel time (see Table 1). The reverse was true for the other measures, indicating a tendency for longer actual routine travel distances among those who live further away from primary roads. However, as 88% respondents in this sample lived within 5 km of a primary road, future work might explore the role of proximity to primary roads with shorter distances.

### Access to primary care opportunities

We defined accessible primary healthcare opportunities (PCOs) as those located within a respondent's activity space. To examine accessibility, we looked at three sets of descriptive statistics for each representation of activity space: the mean and median number of primary care opportunities within an individual's activity space, the percent of respondents with at least one primary care opportunity inside their activity space, and correlation between the area of activity space and the number of primary care opportunities (Table 3).

**Table 3:** Access to primary care opportunities for the five activity space measures. Spearman's rank correlation. SDE1 = standard deviational ellipse at 1 standard deviation; SDE2 = standard deviational ellipse at 2 standard deviations; RNB = road network buffer; STT = standard travel time polygon; RTT = relative travel time polygon.

	**Measure of activity space**									
	
	**SDE1**		**SDE2**		**RNB**		**STT**		**RTT**	
	Mean (S.D.)	Median (Range)	Mean (S.D.)	Median (Range)	Mean (S.D.)	Median (Range)	Mean (S.D.)	Median (Range)	Mean (S.D.)	Median (Range)

**Number of primary care opportunities**	6.04 (9.47)	3 (0–104)	13.71 (18.68)	8 (0–155)	13.06 (12.90)	9 (0–86)	39.59 (37.55)	21 (0–142)	39.33 (44.55)	20 (0–232)
										
**Percent with at least one primary care opportunity**	77.6		92.8		97.5		99.9		99.0	
										
**Correlation between area and number of primary care opportunities**	0.426		0.506		0.441		0.787		0.790	

The distribution of the number of PCOs was skewed to the right, like the area measures. The SDE1 – the activity space measure with the smallest median area – also had the smallest median number of PCOs (3). The median number of PCOs was highest for the largest measures, STT (21) and RTT (20). The RNB (9) and SDE2 (8) were similar in spite of the larger area of the SDE2; because the SDE2 captures 95% of all activity destinations, the similarity in number of PCOs with the RNB measure (which captures 100% of activity destinations) is perhaps not surprising.

All but one respondent had at least one primary care opportunity inside the STT activity space, thus one respondent lacked access according to the federal maximum travel time of 30 minutes to a primary care provider. It is worth noting that this respondent's measured travel time to the nearest primary care opportunity was 30.52 minutes. Although at the limit of the 30-minute travel time standard, this respondent's threshold travel time for the RTT measure was 92 minutes, and the same respondent's RTT polygon had 134 primary care opportunities inside.

The range of opportunities was greatest for the RTT (0–232). Only 11 respondents lacked access using the RTT measure; of these, all had very small RTT areas, ranging from 0.04–136 square kilometers (all in the lowest quartile for area). However, all of these respondents did have primary care opportunities within their STT activity space (range of 6–52). The RNB and SDE2 also had a surprisingly high percentage with at least one primary care opportunity (97.5% and 92.8% respectively). Only the SDE1 measure indicated a substantial percentage of respondents without access to a primary care opportunity (22.4%).

Spearman's rank correlation was performed to test the strength of association between the area of activity space and the number of primary care opportunities for each activity space model. While each activity space model demonstrated a positive correlation between the area and the number of opportunities, the association was strongest for the STT (.787) and RTT (.790). This was not necessarily expected, given the weak negative correlation between the two area measures.

### Correspondence between actual healthcare destinations and activity space

In order to assess which of the activity space measures best captures the health travel behavior of the respondents, we compared the proportion of respondents whose actual health destinations are captured by each measure (Table 4). The two largest area measures, STT and RTT, capture the health destinations of the highest percentage of respondents (86% and 82%, respectively), while the SDE1 captured the fewest. The SDE2 captured slightly more actual health destinations than the RNB. The RNB measure captured 59% of actual health destinations, indicating that in this sample, the majority did choose providers located near their routine activity destinations or along the assumed path of their travel to these destinations.

**Table 4:** Correspondence of actual healthcare destination with area and number of primary care opportunities. Among respondents reporting at least one visit to a health care provider in the past year and providing locational data for a provider (n = 807). Spearman's rank correlation. SDE1 = standard deviational ellipse at 1 standard deviation; SDE2 = standard deviational ellipse at 2 standard deviations; RNB = road network buffer; STT = standard travel time polygon; RTT = relative travel time polygon.

	**Activity space measures**				
	**SDE1**	**SDE2**	**RNB**	**STT**	**RTT**

**Percent of respondents whose reported health destination is inside the activity space**					
Total	40.2	62.3	58.6	86.1	81.7
					
By Area of Activity Space (quartiles)					
Smallest – 1^st^	22.3	47.7	46.2	77.9	58.3
2^nd^	37.3	57.9	55.8	86.6	83.6
3^rd^	44.2	69.5	70.4	87.8	88.5
4^th^	57.1	75.7	61.9	93.0	95.6
					
By Number of Primary Care Opportunities (quartiles)					
Fewest – 1^st^	13.5	39.0	29.9	58.7	44.4
2^nd^	36.3	58.5	46.4	64.5	63.2
3^rd^	51.3	71.9	50.8	68.5	70.1
4^th^	67.0	80.9	54.7	74.6	75.1
					
**Correlations**					
between health destination inside and area of activity space	.267	.242	.152	.166	.343
					
between heath destination inside and number of primary care opportunities	.496	.360	.241	.219	.362

The percent of respondents with a health destination inside increased significantly by quartile of area for all measures. For example, the percentage with a health destination inside increased from 58% to 96% from the lowest to highest quartile for the RTT measure. Area was mildly correlated with having a health destination inside the activity space, with the RTT measure having the strongest association (.343) and the RNB the weakest (.152).

Positive correlations were found between having a health destination inside the activity space and the number of PCOs inside the activity space (Table 4). The strongest correlation was with the SDE1 measure (.496) and the weakest with the STT measure (.219). Again, the percentage increased significantly by quartile of number of PCOs. For the SDE1 measure, 14% had a health destination inside at lowest quartile of number of PCOs; this increased to 67% for the highest quartile.

Table 5 shows the results of logistic regression testing the association between area and the success of the activity space in capturing the actual health destination. Unadjusted odds ratios show the strongest effect of area of the RTT (OR = 3.37) and the weakest for the RNB (OR = 1.35). Adjusted odds ratios show the association with area after controlling for the number of primary care opportunities; although the effect of area is reduced, the area of the RTT (OR = 2.33) again has the strongest effect of the five area measures.

**Table 5:** Predicted odds of the health destination being located inside the activity space by area, adjusted for number of primary care opportunities. Area and Number of SDP measures truncated above 98^th ^percentile to the 98^th ^percentile. Area measures square root transformed. Area measures, counts of primary care opportunities normalized using Z scores and centered around the mean. SDE1 = standard deviational ellipse at 1 standard deviation; SDE2 = standard deviational ellipse at 2 standard deviations; RNB = road network buffer; STT = standard travel time polygon; RTT = relative travel time polygon.

	**Unadjusted odds ratio**	**CI**	**Adjusted odds ratio**	**CI**
**Area**				
SDE1	1.75	1.50, 2.05	1.41	1.17, 1.68
SDE2	1.75	1.47, 2.09	1.40	1.14, 1.72
RNB	1.35	1.17, 1.56	1.18	1.01, 1.39
STT	1.68	1.37, 2.07	1.21	0.92, 1.59
RTT	3.37	2.51, 4.52	2.33	1.57, 3.46

## Discussion

Activity space is a measure of where people go on a routine basis. Mapping a person is different from mapping a stationary object – people are not fixed to a single location, and so cannot adequately be represented with a fixed-location, one-dimensional point. By obtaining locations of routine destinations, a two-dimensional measure of activity space can be developed to represent the space a person occupies as they perform the routines of daily life. The increasing use of GIS in accessibility studies has not only made activity space a viable alternative to distance as a measure of geographic accessibility, but has increased the methodological options available to researchers to represent activity space and measure accessibility. This paper compares five of these options; a summary of each measure is provided in Table 6.

**Table 6:** Summary of activity space measures. SDE1 = standard deviational ellipse at 1 standard deviation; SDE2 = standard deviational ellipse at 2 standard deviations; RNB = road network buffer; STT = standard travel time polygon; RTT = relative travel time polygon.

	**Representation of accessibility**	**Type and shape of measure**	**Data sources**	**Advantages**	**Disadvantages**	**Specific applications**
**SDE1** **SDE2**	Statistical approximation; abstract space	Euclidean measure; ellipsoid shape	Household + activity locations (multiple points), frequency-weighted	Captures spread and orientation of points; can be weighted by frequency	Capture 67% and 95% of points, respectively; Euclidean measures do not fully capture surface effects; poor representation of actual activity space; captures opportunities not in activity space; requires a minimum of three unique points to generate ellipse	SDE1 has statistical/predictive power (in this study area)
**RNB**	Descriptive, Actual access	Network-based measure; shape is buffered network ("worm")	Household + activity locations (multiple points), road network	Captures 100% of activity destinations – best representation of actual space determined by nodes and routes	May be too restricted for predictive purposes	Bypassing; accessibility in actual activity space
**STT**	Normative, potential access, single norm (30-minute travel time threshold)	Network-based measure; shape is network-derived polygon	Household (single point), road network, travel time standard	Results are fundamentally different from other measures; captures the highest number of SDPs; area is indicator of relative location within road network	Not based on activity destinations; "arbitrary" travel time limit? Most strongly conditioned by location relative to road network	Evaluate accessibility according to standards/guidelines. Area is indicator of proximity to primary roads, services
**RTT**	Normative, potential access, relative norm (individually determined)	Network-based measure; shape is network-derived polygon	Household (single point), road network individual travel time threshold (maximum travel time to routine activity destination)	Captures 100% of activity destinations; highly correlated with RNB (can be used in combination)	May overlap with STT; also conditioned by location within road network hierarchy	Relative accessibility; area has strongest correlation with health destination

The results support conclusions made by others that there is no one "best" measure for accessibility, that different measures capture or emphasize different dimensions of accessibility, and that the question being asked should determine the appropriate selection of measure [10,46]. The different measures provided different results when asking the same question. A primary care opportunity could appear "accessible" using the SDE1 but not when using the RNB, or vice-versa. The different activity space measures captured different numbers of primary care opportunities, providing a different assessment of accessibility. To the extent that a single measure can be considered the "best" measure to study accessibility, the question or problem to be solved largely determines the most appropriate measure. For example, the RNB measure might be the best approach for studying bypassing behavior, as the measure is the best representation of actual activity space. Different distance thresholds or other accessibility parameters can be established to measure potential access, as we did with the 30-minute travel time standard STT polygon. And RTT combines the descriptive with the normative by using observed distance tolerances to describe individual potential access; results demonstrate the value of this perspective. And the SDE remained valuable – the SDE2 often produced similar results as the other measures; yet given the high level of accessibility in this region, the more discriminating SDE1 may have more statistical power.

The STT measure was distinct from the other measures. The results presented here correspond with Kwan's finding of weak correlations between space-time measures and integral measures and her conclusion that the two types of measures are distinctive and "capture different dimensions of the accessibility experience of individuals" [10]. One interesting consequence of using the 30-minute travel time polygon was how the road network structured the results. Proximity to primary roads influenced the area of the STT activity space and its association with healthcare accessibility in two ways. First, visual analysis of the data confirmed that the primary care opportunities tend to be located on primary roads and clustered around the intersection of two or more primary roads, so that in general, respondents who lived nearer to primary roads also lived nearer to more service delivery points. Second, with higher speed limits assigned to primary roads, respondents who lived nearer to primary roads can travel longer distances in a 30-minute time span and have larger potential activity spaces, which then contain a higher number of primary care opportunities. Users of this type of measure should recognize how it represents this specific dimension of accessibility – proximity to primary roads, or more specifically, how it is conditioned by (data on) road type and speed limits. The contrasting trends between the STT and the other measures suggest that while those who live near primary roads can travel greater distances, they also live closer to services and other opportunities and thus tend to have smaller activity spaces. Conversely, those who live farther away from primary roads cannot travel as far within a given a given travel time limit, however they routinely travel farther to routine activities, and tend to have larger activity spaces. While it may seem obvious, this dynamic has not typically been recognized in discussions of activity space or accessibility.

Assumptions about the mode of transportation become very important, as not all respondents have the same access to a private vehicle. As reported elsewhere [47], 13% of respondents did not have a driver's license and approximately 5% of respondents had used public transportation to attend a healthcare visit in the year preceding the interview. In the U.K., GIS has been used to model travel times by various modes of public and private transportation [48], however, the public transportation system is much more well developed in the U.K. than in the U.S., let alone rural America. Developing alternative distance thresholds for persons who do not have access to a car or are unable to drive – situations with specific relevance for regions with high levels of poverty or high proportions of retired and elderly populations – may be useful to better approximate the activity space for those without access to a private automobile.

Although the goal of the paper was primarily methodological, the healthcare context of this region nonetheless influenced our results. The study site was selected for its relative isolation and sparse population density, as well as hypothesized of gaps in healthcare accessibility for some segments of the population; we instead found that the study area had both a well-developed transportation corridors and supply of primary care services [38]. Given the small numbers of respondents who lived more than 20 minutes from a primary care service delivery point, we were unable to compare those with poor access to those with good access (as defined by the 30-minute travel time standard).

While we believe that activity space as a conceptual model has the potential to become a more widely utilized tool in studies of spatial access, we do not wish to overstate the ease of incorporating these methods into many research projects. One of the unique strengths of this work is the rich source of activity data. Having the respondents' home locations as well as the locations and frequency of their activity destinations enabled us to create a reasonably complete activity space. The data and methods section details the data collection effort required for constructing activity spaces, and this level of effort may remain beyond the resources of many researchers. The activity data was only one part of a very long, in-depth survey that took up to two hours to complete and involved the payment of respondents for their time. Moreover, the project was able to contract with a professional research firm to implement the survey. In addition to the survey data, the project was fortunate to acquire a database of healthcare providers for the region that was compiled contemporaneously and had been georeferenced. This conjunction of detailed datasets, as well as access to some higher-end network analysis tools, afforded us the unique opportunity to create and analyze these models of activity space. Unfortunately, given the cost of datasets such as StreetMap 2000, the paucity of georeferenced health services databases, and the data collection requirements, others may not be able to fully duplicate the methods described.

On the other hand, these measures are relatively simple to construct when compared with the models described by Golledge and Stimson [12], or the algorithms that Kwan and colleagues employ to construct space-time measures [9,10,49]. There are tradeoffs between the specificity detailed in 2-day travel diaries and the more spatially- and temporally-generalized measures we constructed. We believe that for the purpose of studying healthcare accessibility, the measures we propose are appropriate and easier to implement; of course this may not be the case for all questions and applications. As desktop applications are developed that simplify the generation of activity spaces [50], the increasing ease of applying these measures will expand the range of potential applications.

## Conclusion

The availability of geospatial technologies and data create multiple options for representing and operationalizing the construct of activity space. Echoing Kwan and colleagues [13], our aim is to advance accessibility research by examining the interrelated issues of method, representation, and application. Each of the five measures represent a methodological variation on a single theoretical construct of activity space. If we define "accessibility" as the availability of healthcare opportunities within that individual's activity space, access is influenced by the shape and area of the activity space, the spatial distribution of opportunities, and by the spatial structures that constrain and direct movement through space. The shape and area of the activity space is influenced by individual factors, spatial structures, and the locations of other opportunities; it is also influenced by how activity space is conceptualized and measured.

The paper demonstrates how activity space can be used to (a) examine the correspondence between location of health services and individual activity spaces, (b) assess the extent of bypassing in accessibility studies, and (c) test a travel time standard from the individual perspective, and (d) compare that standard to actual travel patterns. The analysis also shows that, in four of the five representations of activity space, the majority of respondents did in fact use a healthcare provider within their activity space, demonstrating the relevance of activity space to healthcare accessibility.

The five measures presented, SDE1, SDE2, RNB, STT and RTT together provide a multi-faceted picture of a respondent's activity space. The first approach, the SDE, provides a theoretical or abstract representation using Euclidean space. It quantifies the size and general orientation of the spread of destinations to which a respondent travels. It uses abstract space to represent material spatial arrangements and is not well suited for more sophisticated analysis looking at issues of bypassing or modeling routes taken by the respondent. The RNB measure is better at these types of analysis than SDE. By creating buffers around the road network its possible to analyze bypassing and know the network distance necessary to reach a destination, something that is important in an area that has physical barriers to straight, Euclidean travel. The two travel time measures, STT and RTT provide a glimpse of another important aspect of a respondent's activity space, namely travel time. The STT approach uses the federal standard of 30 minutes to determine access to primary care opportunities for each respondent. This can provide a picture of the degree of underservice for the respondent, or more generally, how they fare against the federal guidelines. The other travel time measure, RTT, is linked to the actual destinations a respondent travels to and provides a picture of the burden of travel to healthcare destinations relative to the burden of other routine activity destinations.

We have also shown that each measure of activity space can help inform the interpretation of the others, and conclude that the use of multiple measures is better than relying on a single measure. Triangulation in the social science context refers to examining a phenomenon from multiple perspectives to gain a more complete and nuanced interpretation of data. The most significant burden in any GIS-based analysis involves the collection of spatial data and the development of the spatial database; the additional burden of constructing and comparing multiple measures is relatively small once the database is established, and the benefits of using multiple measures outweigh the additional costs.

## List of abbreviations

CFCC Census Feature Class Codes

GIS Geographic Information Systems

GISc Geographic Information Science

GPS Geographic Positioning System

I-26 Interstate 26

I-40 Interstate 40

I-85 Interstate 85

MAP Mountain Accessibility Project

MSA Metropolitan Statistical Area

PC Primary Care

PCO Primary Care Opportunity

RTT Relative Travel Time

RNB Road Network Buffer

SD Standard Deviation

SDE Standard Deviational Ellipse

SDE1 Standard Deviational Ellipse at 1 Standard Deviation

SDE2 Standard Deviational Ellipse at 2 Standard Deviations

STT Standard Travel Time

## Authors' contributions

JES contributed to the study design, performed the statistical analysis, and drafted the manuscript. JS contributed to the study design, constructed the spatial measures and performed the spatial analysis, and helped to draft the manuscript. JSP contributed to the overall project design, oversaw data management and statistical analysis, and critically contributed to the manuscript. WMG conceived of the overall project of which this study is a part, assisted in the management of the data collection, and critically contributed to the manuscript. TAA assisted in the design of the overall project, managed data collection, and critically contributed to the manuscript.
